# Next-generation pathogen genomics

**DOI:** 10.1186/s13059-014-0528-6

**Published:** 2014-11-19

**Authors:** George M Weinstock, Sharon J Peacock

**Affiliations:** The Jackson Laboratory for Genomic Medicine, Farmington, Connecticut USA; Department of Medicine, University of Cambridge, Box 157 Addenbrooke’s Hospital, Hills Road, Cambridge, CB2 0QQ UK; The Wellcome Trust Sanger Institute, Wellcome Trust Genome Campus, Hinxton, Cambridge CB10 1SA UK

## Editorial

In the early 1990s, one of us was involved in one of the first projects to sequence a bacterial genome, the meager 1.1 Mb chromosome of *Treponema pallidum*, the causative agent of syphilis. Completing the project ultimately took about seven years (until published in 1998 [1]), over US$1.8 million in National Institutes of Health grants (R01AI031068 and R01AI040390) [2], and required pooling forces with The Institute for Genomic Research. Recently, that original *T. pallidum* strain was re-sequenced to get a ‘perfect’ sequence, a process that took a few days and cost only hundreds of dollars [3]. The original sequencing was performed with the dideoxy-chain termination technique using slab gel electrophoresis instruments. Newly developed software was used for genome assembly and data management and analysis. The latter re-sequencing was performed with next-generation sequencing (NGS) technology and mature software tools. Such is the enormous progress in microbial genome sequencing in the last 20 years.

The mind-boggling evolution of DNA sequencing and bioinformatics technologies is driving a new era of pathogen research. Recent studies of old, well-scrutinized pathogens are now greatly extended based on the sequencing of thousands of strains from collections [4,5]. This increased density of genetic data for individual species allows new insights and definition of mechanisms, just as an aerial photograph gives a clearer picture of the landscape as the pixel density increases. Such large-scale studies, now possible with the increased throughput and lower cost of sequencing, allow a more comprehensive picture of a species’ gene pool (the pan-genome), population genetic and/or evolutionary analyses, and more accurate insights into epidemiology, to name a few advances. In the realm of epidemiology, NGS of pathogens is now pushing into the applied genomics area of the clinic, with, for example, studies of clinical outbreaks that can now precisely define complex transmission chains [6,7]. Perilous clinical challenges posed by new antibiotic-resistant organisms benefit from NGS which can identify mutations, thereby defining mechanisms by which resistance is acquired [8,9], as well as discerning new threats from resistance genes found in whole genome sequences [10].

It is in this context of a new era in pathogen genomics that this special issue of *Genome Biology* and *Genome Medicine* on the Genomics of Infectious Diseases has been assembled. It coincides with an exhilarating time for pathogen genomics research and covers a broad range of bacterial, viral, and parasitic pathogens. Genomic analysis, and sequencing in particular, is agnostic, and applies equally well to the diverse types of pathogens studied in this special issue. Pathogen genomics continues to be an area of some urgency. We need look no further than the current challenges of containing Ebola virus outbreaks or the emergence and expansion of new antibiotic-resistant bacteria, such as carbapenemase-producing *Klebsiella pneumoniae*, to be reminded that infectious disease is not, and will never be, a solved problem. Rather, only by dramatic technological innovation, such as offered by NGS, can we keep up with the pathogenic population.

Genome sequencing continues to advance and provide new tools and applications for pathogen research. Sequencing can now be performed on hundreds of strains in parallel in overnight instrument runs, and this drives forward the data density for the description of genomes and gene expression patterns. Metagenomic application of NGS is another bright new area, affording new culture-independent detection of pathogens in clinical samples as well as illuminating interactions between the pathogen and resident microbiome. One looks forward to future applications of this information to combat infection and restore health, possibly with reduced dependence on antibiotics.

## Abbreviation

NGS: Next-generation sequencing
